# Survival Benefits of GLP-1 Receptor Agonists in Patients with Neuroendocrine Neoplasms: A Large-Scale Propensity-Matched Cohort Study

**DOI:** 10.3390/cancers17091593

**Published:** 2025-05-07

**Authors:** Manal S. Fawzy, Awwad Alenezy, Jessan A. Jishu, Issa Khan, Ahmad Dessouky, Ahmed Abdelmaksoud, Kristen E. Limbach, Eman A. Toraih

**Affiliations:** 1 Center for Health Research, Northern Border University, Arar 73213, Saudi Arabia; manal.darwish@nbu.edu.sa; 2 Department of Family and Community Medicine, Faculty of Medicine, Northern Border University, Arar 91431, Saudi Arabia; 3 School of Medicine, Tulane University, 1430 Tulane Ave., New Orleans, LA 70112, USA; jjishu@tulane.edu; 4 SUNY Upstate Medical University, New York, NY 13210, USA; 5 Faculty of Medicine, Suez Canal University, Ismailia 41522, Egypt; 6 Department of Internal Medicine, University of California, Riverside, CA 92521, USA; 7 Department of Surgery, School of Medicine, Tulane University, 1430 Tulane Ave., New Orleans, LA 70112, USA; klimbach@tulane.edu; 8 Department of Cardiovascular Perfusion, Interprofessional Research, College of Health Professions, SUNY Upstate Medical University, New York, NY 13210, USA; 9 Genetics Unit, Department of Histology and Cell Biology, Faculty of Medicine, Suez Canal University, Ismailia 41522, Egypt

**Keywords:** neuroendocrine tumor, neuroendocrine carcinoma, NEN, NET, NEC, semaglutide, tirzepatide, diabetes, obesity, lung cancer, PNET, pancreatic cancer

## Abstract

**Background:** Neuroendocrine neoplasms (NENs) represent a heterogeneous group of malignancies that consist of two major subtypes: neuroendocrine tumors (NETs) and neuroendocrine carcinomas (NECs). Glucagon-like peptide-1 receptor agonists (GLP-1Ra) have demonstrated favorable results in preclinical studies, but their impact on NEN outcomes remains unexplored. **Methods:** Using the TriNetX US Research Network, we identified adult patients with NEN and either diabetes or obesity. After 1:1 propensity score matching based on demographics, comorbidities, procedures, and medication use, we compared survival outcomes between patients who received GLP-1Ra after NEN diagnosis and those who did not. **Results:** Among 32,464 eligible patients, 3139 received GLP-1Ra and 29,325 did not. After propensity matching, each cohort included 3043 patients with well-balanced baseline characteristics. During follow-up periods extending up to 15 years, all-cause mortality occurred in 356 (11.7%) GLP-1Ra users versus 753 (24.7%) non-users, representing a 13.0% absolute risk reduction (*p* < 0.001). GLP-1Ra use was associated with significantly improved survival (HR = 0.56, 95%CI = 0.49–0.63, *p* < 0.001). Both well-differentiated (HR = 0.52) and poorly differentiated tumors (HR = 0.56) showed significant improvement. Among primary sites, lung NENs demonstrated the most pronounced benefit (HR = 0.42). Tirzepatide showed the strongest association with reduced mortality (HR = 0.16), followed by semaglutide (HR = 0.27) and dulaglutide (HR = 0.52). **Results:** In this large propensity-matched study, GLP-1Ra use was associated with a 44.3% reduction in mortality risk among NEN patients with diabetes or obesity. The magnitude of the observed benefit suggests a potential role for GLP-1Ra as adjunctive therapy in this patient population. Prospective clinical trials are warranted to confirm these findings and explore underlying mechanisms.

## Simple Summary

Glucagon-like peptide 1 receptor agonists (GLP-1Ra) are a popular class of medication that has been shown to reduce cancer risk, but their effects on patients with neuroendocrine neoplasms (NENs) have not yet been studied. We assessed their effects on patients with NENs and showed that they may improve survival in these cancer patients. These medications show great promise and may assist physicians in treating their patients with this disease.

## 1. Introduction

Neuroendocrine neoplasms (NENs) represent a heterogeneous group of tumors that arise from enterochromaffin cells, with characteristics of hormone-producing cells. These tumors develop across various anatomical sites but are most common in the gastrointestinal tract, lungs, and pancreas [1,2]. The 2022 WHO Classification of Endocrine and Neuroendocrine Tumors has classified them into two general categories: well-differentiated neuroendocrine tumors (NETs) and poorly differentiating neuroendocrine carcinomas (NECs) [3,4]. While NETs often follow a less aggressive clinical course, their invasive and metastatic potential is influenced by factors such as tumor grade, site of origin, and receptor expression profiles [5]. Despite accounting for only about 0.5% of all malignancies, the incidence of NET has increased more than five-fold over the past four decades [6,7]. This rising prevalence, combined with often prolonged disease courses, presents significant challenges for clinicians and healthcare systems.

The heterogeneity of NEN presentation caused by diverse primary sites, a spectrum of behavior ranging from indolent to aggressive, and functional statuses complicates treatment optimization [8]. Surgical resection continues to be the primary curative approach for localized disease, while management of advanced NEN typically involves a combination of surgical cytoreduction, somatostatin analogs, targeted therapies, and peptide receptor radionuclide therapy [9,10].

The glucagon-like peptide-1 receptor (GLP-1R) has emerged as a focus of interest in both diabetes and oncology research [9]. GLP-1, an endogenous incretin hormone, binds to GLP-1R on pancreatic beta cells, enhancing glucose-dependent insulin secretion and lowering blood sugar [11]. Additionally, GLP-1 acts on receptors in the brain and stomach to inhibit gastric emptying, acid secretion, and motility, which collectively decrease appetite and promote satiety [12]. GLP-1 agonists (GLP-1s), including semaglutide, liraglutide, dulaglutide, and tirzepatide, are exogenous incretin mimetics that can bind to GLP-1R independently of food intake, effectively managing diabetes and promoting weight loss in obesity.

GLP-1R deregulation has been identified in various cancer types, including thyroid cancer, pancreatic adenocarcinoma, and breast cancer [13,14,15]. Notably, GLP-1R expression has been documented in certain NENs, particularly pancreatic NETs, raising questions about the potential impact of GLP-1Ra therapy on tumor behavior and survival outcomes in this patient population [16]. Despite promising preclinical findings regarding the reduction in tumor growth through hormonal mechanisms, the clinical impact of GLP-1Ra on cancer outcomes, particularly in NEN, remains largely unexplored [17]. While small observational studies have reported mixed results regarding associations between GLP-1Ra use and cancer progression in patients with various malignancies, no large-scale study has specifically examined the relationship between GLP-1Ra treatment and survival outcomes in patients with NEN.

To address this critical knowledge gap, we conducted a large propensity-matched cohort study using the TriNetX database to investigate whether GLP-1Ra use has an impact on survival in patients with NEN. This study could provide valuable insights into the potential repurposing of widely prescribed metabolic agents as adjunctive treatments for NEN with implications that could shape both current clinical approaches and future clinical trial design.

## 2. Materials and Methods

### 2.1. Database Characteristics

We utilized the TriNetX database (US Collaborative Network), which includes de-identified electronic medical records from 67 healthcare organizations (HCO). This database contains longitudinal clinical data on demographics, diagnoses, procedures, medications, laboratory values, and mortality data spanning the past 20 years (accessed on 14 March 2025).

### 2.2. Study Population

We identified adult patients (aged 18 years or more) diagnosed with NEN (ICD-10 code C7A) who also had a documented diagnosis of diabetes mellitus (ICD-10 codes E08-E13) or obesity (ICD-10 code E66). NEN patients were categorized into two groups: (1) patients who received at least one prescription for GLP-1Ra analogs (ATC code A10BJ) or tirzepatide (RXNORM code 2601723) within one year after NEN diagnosis, and (2) those who never received GLP-1Ra therapy. The primary outcome was all-cause mortality.

### 2.3. Propensity Score Matching Analysis

To balance covariates in both cohorts and minimize confounding, we performed 1:1 propensity score matching using nearest-neighbor matching. The propensity model included demographic factors (age at index, sex, race), comorbidities (types 1 and 2 diabetes mellitus, obesity, primary hypertension, cerebrovascular diseases, acute and chronic kidney disease), procedures (surgery, radiation therapy, complex drug administration), and medication use (antineoplastics). Matching was assessed using standardized mean differences, with values < 0.1 indicating adequate balance.

### 2.4. Stratification

We conducted pre-specified subgroup analyses to assess potential effect modification. Stratification variables included demographic factors (age < 65 vs. ≥65 years; male vs. female), tumor characteristics (well-differentiated NEN [ICD-10-CM: C7A.0] vs. poorly differentiated NEN [ICD-10-CM: C7A.1]), primary tumor location (gastrointestinal [ICD-10-CM: C7A.01*, C7A.02*, C7A.092], pancreatic [ICD-10-CM: C25.4 in combination with C7A.*], and lung [ICD-10-CM: C7A.090]), and specific GLP-1Ra medication used (semaglutide, liraglutide, dulaglutide, or tirzepatide).

### 2.5. Statistical Analysis

We used built-in analytics tools in the TriNetX platform and R for data analysis. A two-sided Chi-square test was employed to compare the two groups. For survival analysis, Cox proportional hazard models were performed to assess time to events. Hazard ratios (HRs) with 95% confidence intervals (CIs) were reported. Kaplan–Meier survival curves were plotted. The two-sided test was used to assess differences in survival curves. Results were assessed before and after propensity score matching analysis. Statistical significance was defined as *p* < 0.05.

## 3. Results

### 3.1. Patient Characteristics

The network included 120,169,374 patients, of which 102,541,692 were adults. We identified 81,838 patients (66 HCO) with NENs. Of these, 32,464 patients (65 HCO) had comorbid diabetes or obesity. The study cohort was further stratified into 3139 patients who initiated GLP-1Ra treatment after NET diagnosis and 29,325 control patients who did not receive GLP-1Ra therapy. After propensity score matching with a 1:1 ratio, the final analysis included 3043 GLP-1Ra users (51 HCO) and 3043 matched controls (65 HCO). Baseline characteristics of the study population are depicted in Table 1.

### 3.2. Follow-Up Time

GLP-1Ra users had a median follow-up of 523 days (interquartile range 847 days) with a mean follow-up of 750.0 days (standard deviation 735.5 days). Non-users had a median follow-up of 664 days (interquartile range 1370 days) with a mean follow-up of 1052.2 days (standard deviation 1066.5 days). For some patients in both cohorts, follow-up periods extended up to approximately 15 years (5500 days).

### 3.3. Overall Survival

**Figure 1:**
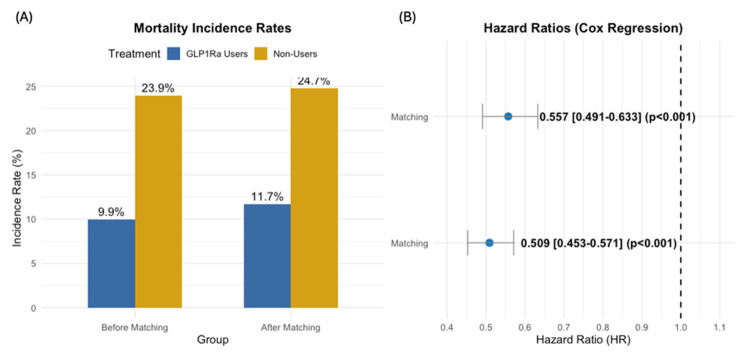
Mortality rates and hazard ratios. (**A**) Mortality incidence rates for GLP-1Ra users (blue) and non-users (gold) before and after propensity score matching showed a lower mortality rate in users. (**B**) Cox regression hazard ratios (HRs) with 95% confidence intervals before and after matching, indicating a significant reduction in mortality risk for GLP-1Ra users. Values less than 1.0 (dashed line) indicate reduced mortality risk with GLP-1Ra treatment.

**Figure 2:**
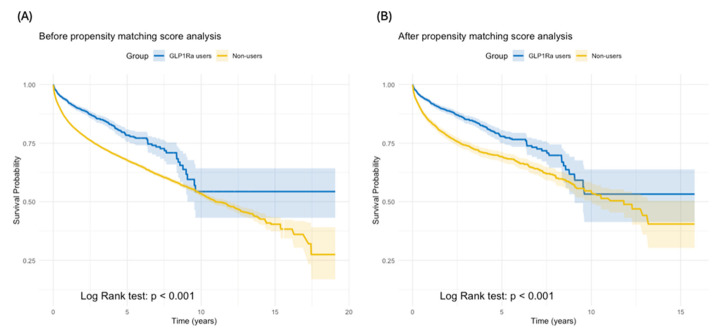
Kaplan–Meier survival curves. (**A**) Survival curves before matching show higher survival for GLP-1Ra users (*p* < 0.001). (**B**) After matching, the survival benefit remains, with significant differences between groups (*p* < 0.001). Shaded areas indicate 95% confidence intervals.

Before propensity matching (GLP-1Ra users n = 3046; non-users n = 28,878), mortality was reported for 303 (9.95%) GLP-1Ra users versus 6906 (23.9%) non-users (*p* < 0.001). GLP-1Ra use was associated with improved survival (HR 0.509, 95% CI: 0.453–0.571). After propensity matching (n = 3043 per cohort), mortality was reported for 356 (11.9%) GLP-1Ra users versus 753 (24.7%) non-users (*p* < 0.001). The survival benefit with GLP-1Ra persisted (HR 0.557, 95% CI: 0.491–0.633) (Figure 1). At the end of follow-up, the survival probability was 53.23% for GLP-1Ra users compared to 40.48% for non-users. Kaplan–Meier curves of all-cause mortality are shown in Figure 2.

### 3.4. Subgroup Analysis

**Figure 3:**
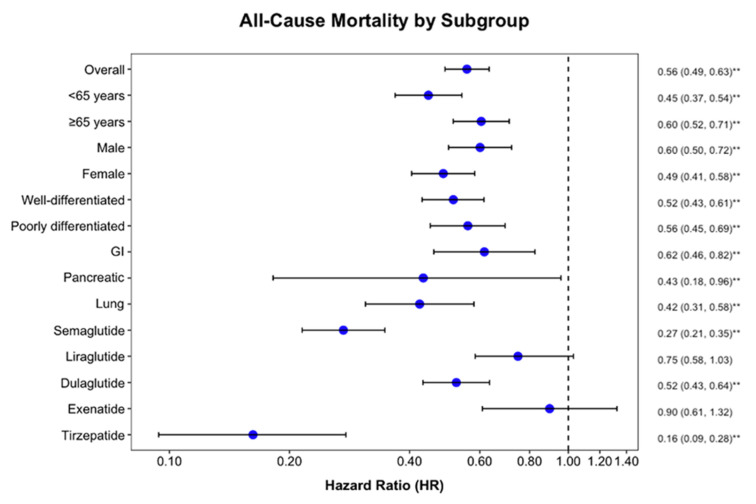
Forest plot showing hazard ratios (HRs) for all-cause mortality among patients with neuroendocrine neoplasms treated with GLP-1Ra compared to propensity-matched controls. Values less than 1.0 (dashed line) indicate reduced mortality risk with GLP-1Ra treatment. ** indicates statistically significant results (*p* < 0.05). GLP-1Ra = glucagon-like peptide-1 receptor agonist.

The survival benefit persisted across age groups, sex, tumor grade, and primary tumor sites (Table 2). Notably, both NETs (HR = 0.52) and NECs (HR = 0.56) showed significant improvement with GLP-1Ra therapy. Among different primary sites, lung NENs demonstrated the most pronounced benefit (HR = 0.42) (Figure 3). Among specific GLP-1Ra agents, tirzepatide showed the strongest association with reduced mortality (HR = 0.16), followed by semaglutide (HR = 0.27) and dulaglutide (HR = 0.52). In contrast, liraglutide (*p* = 0.12) and exenatide (*p* = 0.91) did not demonstrate statistically significant survival benefits.

## 4. Discussion

Although it is well known that GLP-1Ra improves prognosis for many conditions, its effects on certain cancers remain a question. For instance, they have been well studied in obesity-related cancers with favorable outcomes, but there are limited studies confirming these effects in non-obesity cancers [18,19]. Moreover, they were noted to have contrasting or controversial effects on thyroid and pancreatic cancers [20]. Given that the incidence and mortality rates of NENs have been increasing over the past two decades, there exists a strong need for further therapeutic options [21]. The present study revealed significantly improved survival among NEN patients receiving GLP-1Ra therapy. After propensity score matching, GLP-1Ra users exhibited 44.3% lower mortality risk versus non-users. This substantial difference persisted across demographic subgroups and tumor types, suggesting therapeutic benefits beyond metabolic management.

**Figure 4:**
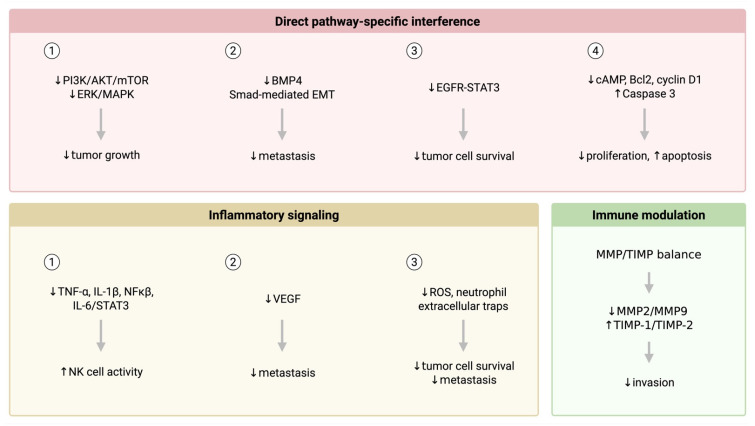
Plausible physiologic and molecular mechanisms of tumor growth suppression by GLP-1Ra medications.

Such observed benefits might arise through several plausible mechanisms (Figure 4). GLP-1Ra medications create systemic metabolic shifts, reducing hyperinsulinemia and subsequently decreasing the availability of growth factors known to promote cancer progression [22]. This may especially be the case for NENs, which express higher concentrations of growth factor receptors than normal cells, with preclinical studies showing that blockade of these receptors results in NEN cell growth inhibition and apoptosis [23,24]. Furthermore, the significant weight and fat reduction associated with GLP-1Ra agents may diminish pro-inflammatory cytokine production from adipose tissue such as IL-6. Such cytokines play significant roles in tumor progression when activating the mTOR signaling pathway, one that is essential for controlling the cell cycle and highly targeted by chemotherapeutic drugs due to its upregulation in NENs [25].

Although we found survival benefits among all NEN types after receiving GLP-1Ra therapy in the present study, our findings contrast with select preclinical and clinical studies demonstrating tumor progression in GLP-1R-expressing pancreatic NENs treated with these agents [26,27]. Cases et al. assessed the significance of GLP-1R expression in 50 resected pancreatic NENs and found that 73% of metastatic lesions stained positive for GLP-1R, although survival was not affected [16]. Butler et al. investigated pancreata resected from 20 individuals and noted that 3 of the 8 who received GLP-1Ra agents were associated with α-cell hyperplasia with the potential to evolve into NENs [28]. Interestingly, there have been suggestions regarding GLP-1R activation inhibiting tumorigenesis and metastasis via the PI3K-Akt signaling pathway, although this was the case for general pancreatic cancer rather than pancreatic NENs [29]. Given the scarcity of published literature in pancreatic NEN and relatively small sample sizes in the available literature, there is a need to study long-term outcomes in patients with this specific tumor.

The differential response observed across specific GLP-1Ra agents warrants attention. We found that tirzepatide and semaglutide demonstrated particularly robust survival benefits (HR 0.16 and 0.27, respectively). This finding partially agrees with Levy et al., who confirmed semaglutide’s excellent cancer-risk reduction across multiple types of cancers [19]. To our knowledge, however, there is no other finding in the literature that demonstrates tirzepatide’s significant survival benefits in any cancer. Indeed, tirzepatide’s dual GIP/GLP-1 activity could explain its superior performance through complementary antitumor pathways. We also found that liraglutide and exenatide showed no significant advantage. These variations might reflect differences in receptor binding affinities, pharmacokinetic profiles, or additional mechanisms beyond GLP-1R activation. Such findings suggest that although GLP-1Ra agents may have a shared primary target to exhibit their effects, individual agents within this class may display peripheral effects, which can influence the risks of overall or specific cancers.

Interestingly, we found that using GLP-1Ra medications induced significant survival benefits in both well-differentiated (NET) and poorly differentiated (NEC) subtypes of NEN in all three primary sites examined (gastrointestinal tract, pancreas, and lung). The latter finding is substantial considering the rarity of NECs, explaining why very little is known about its characteristics and ideal treatment [30,31]. However, it is well known that it has a very poor prognosis despite treatment. The improved survival in NEC can be due to several reasons, especially the anti-proliferative effects within the tumors themselves due to the altered glucose metabolism as a result of these medications. Indeed, this is not surprising given that most cancers elicit hyperglycemia, which have shown to worsen patient outcomes [32]. Furthermore, it may also be due to the possible synergy between these medications and chemotherapy or targeted therapies, although this has only been shown in non-neuroendocrine neoplasms. Nevertheless, we introduce GLP-1Ra medications as promising options for both well-differentiated and poorly differentiated NENs.

Unfortunately, we were unable to assess the impact of tumor stage when assessing the effects of GLP-1Ra medications on NENs. Generally speaking, early NENs can be treated with surgery with the intention to cure while advanced NENs can be treated with debulking techniques and management to reduce tumor burden and improve symptoms [33,34]. As such, it can be argued that GLP-1Ra medications may be more effective in late-stage NEN where surgery may not be beneficial. On the other hand, these medications may also be helpful in early-stage NEN where early initiation of any treatment may be beneficial. The best treatment depends on a plethora of features, which include stage, grade, and primary tumor site. Although more studies are needed to assess the impact of stage on the medications’ influence on NEN prognosis, the ideal treatment is always tailored to the patient and requires careful assessment on the patient’s tumor characteristics.

Although these findings are compelling, our study has limitations that require acknowledgment. The shorter median follow-up time for GLP-1Ra users (523 vs. 664 days) raises important points about potential immortal time bias. For instance, those taking GLP-1Ra medications may have survived long enough after diagnosis to initiate therapy or possess inherent prerequisites that may have attributed to their longer survival. Despite careful matching, residual confounding remains possible, evidenced by the slightly higher antineoplastic medication use among GLP-1Ra recipients. There was also a lack of information on tumor grade in the well-differentiated tumor cohort. Furthermore, information on time from diagnosis to treatment initiation as well as medication adherence, route, dosing, and duration was not available, which limited our ability to assess dose–response relationships or treatment duration effects. Moreover, although we were able to assess overall mortality, we were unable to assess disease-specific mortality, which could provide clarity on the cause of death. Additionally, our reliance on all-cause mortality rather than NEN-specific endpoints, while methodologically sound, may obscure precise mechanisms of benefit.

For clinicians managing patients with both NEN and metabolic disorders, our findings suggest potential dual benefits from GLP-1Ra therapy. This has important clinical relevance since obese and diabetic populations have been reported to have higher prevalences of NENs, implicating that such a treatment may be even more accessible and appropriate for these unique cohorts [35,36,37,38]. However, treatment decisions should still primarily follow established diabetes or obesity guidelines, with potential oncologic advantages considered supplementary until confirmatory evidence emerges. The substantial mortality reduction observed compares favorably with many standard NEN therapies, highlighting a promising avenue for adjunctive treatment. Moving forward, prospective randomized trials stratifying participants by tumor characteristics and GLP-1Ra agent are essential to establish causality and optimize treatment selection. Translational research examining GLP-1R expression patterns across NEN subtypes could identify patients most likely to benefit. Investigation into potential synergies between GLP-1Ra medications and established NET therapies might reveal enhanced treatment strategies.

## 5. Conclusions

To our knowledge, this is the first study to investigate individual GLP-1Ra agents and propose them as treatment options for patients with well-differentiated and poorly differentiated subtypes of NENs. With these medications rising in popularity and usage, their potential application for NEN management offers a practical and accessible therapeutic avenue. Ultimately, this study represents a significant step in understanding the complex relationship between metabolic therapies and cancer outcomes. While mechanistic questions remain, the consistent, substantial survival benefit observed across subgroups provides compelling evidence for further clinical and scientific exploration of GLP-1Ra agents in treating NENs. As these medications continue to gain prominence in clinical practice, exploring their role in NENs could provide an innovative approach for improving patient outcomes with little disruption to current treatment plans.

## Figures and Tables

**Table 1:** Baseline characteristics of NEN patients before and after propensity score matching.

Characteristic	Before Matching			After Matching		
	GLP-1Ra Users (*n* = 3046)	Non-Users (*n* = 28,878)	*p*-Value	GLP-1Ra Users (*n* = 3043)	Non-Users (*n* = 3043)	*p*-Value
Demographics						
Age, mean (SD)						
Age at index, years	62.4 (11.1)	64.2 (12.8)	<0.001	62.4 (11.0)	62.3 (12.1)	0.62
Sex, n (%)			<0.001			0.48
Female	1737 (57.0)	14,438 (50.0)		1735 (57.0)	1765 (58.0)	
Male	1192 (39.1)	13,109 (45.4)		1191 (39.1)	1174 (38.6)	
Race, n (%)			0.22			0.06
White	2132 (70.0)	19,815 (68.7)		2129 (70.0)	2200 (72.3)	
Black/African American	484 (15.9)	4604 (16.0)		484 (15.9)	470 (15.4)	
Asian	74 (2.4)	734 (2.5)		74 (2.4)	57 (1.9)	
Diagnosis, n (%)						
Type 2 diabetes mellitus	2419 (79.4)	11,136 (38.6)	<0.001	2416 (79.4)	2425 (79.7)	0.77
Type 1 diabetes mellitus	350 (11.5)	1190 (4.1)	<0.001	348 (11.4)	325 (10.7)	0.34
Obesity	1949 (64.0)	9587 (33.2)	<0.001	1946 (64.0)	1946 (64.0)	1.00
Primary hypertension	2496 (81.9)	18,031 (62.5)	<0.001	2493 (81.9)	2480 (81.5)	0.66
Cerebrovascular diseases	450 (14.8)	3340 (11.6)	<0.001	450 (14.8)	402 (13.2)	0.07
Kidney disease *	993 (32.6)	6071 (21.0)	<0.001	993 (32.6)	924 (30.4)	0.06
Procedures, n (%)						
Surgery	2669 (87.6)	20,395 (70.7)	<0.001	2666 (87.6)	2646 (87.0)	0.44
Radiation therapy	167 (5.5)	1640 (5.7)	0.64	167 (5.5)	138 (4.5)	0.09
Chemotherapy	265 (8.7)	2143 (7.4)	0.011	265 (8.7)	236 (7.8)	0.053
Medication, n (%)						
Antineoplastics	701 (23.0)	5099 (17.7)	<0.001	698 (22.9)	639 (21.0)	0.07

* Acute kidney failure and chronic kidney disease. Two-sided Chi-square or Student’s *t*-tests were used.

**Table 2:** Case-fatality rates stratified by patient characteristics and GLP-1Ra use in propensity-matched NEN cohorts.

Subgroup	Categories	Count per Group	GLP-1Ra Users	Non-Users (Controls)	*p*-Value
Overall	Overall	3043	356 (11.7%)	753 (24.7%)	<0.001
Age	<65 years	1838	144 (7.8%)	403 (21.9%)	<0.001
	≥65 years	1508	228 (15.1%)	442 (29.3%)	<0.001
Sex	Male	1228	180 (14.7%)	345 (28.1%)	<0.001
	Female	1814	166 (9.2%)	424 (23.4%)	<0.001
Type of NEN	NET	1885	173 (9.2%)	429 (22.8%)	<0.001
	NEC	524	129 (24.6%)	234 (44.7%)	<0.001
Primary tumor site	GI	865	66 (7.6%)	159 (18.4%)	<0.001
	Pancreatic	65	10 (15.4%)	23 (35.4%)	0.009
	Lung	563	54 (9.6%)	154 (27.4%)	<0.001
GLP-1Ra analogues	Semaglutide	1636	83 (5.1%)	425 (26%)	<0.001
	Liraglutide	644	115 (17.9%)	137 (21.3%)	0.12
	Dulaglutide	1199	156 (13%)	330 (27.5%)	<0.001
	Exenatide	211	52 (24.6%)	51 (24.2%)	0.91
	Tirzepatide	592	15 (2.5%)	156 (26.4%)	<0.001

Data are presented as a percentage. A two-sided Chi-Square test was employed to compare between users and non-users within each stratum. NEN: neuroendocrine neoplasm; NET: neuroendocrine tumor; NEC: neuroendocrine carcinoma; GI: Gastrointestinal; GLP-1Ra: glucagon-like peptide-1 receptor agonist.

## Data Availability

Publicly available datasets were analyzed in this study. These data can be found at https://trinetx.com.
